# GYS1 induces glycogen accumulation and promotes tumor progression via the NF-κB pathway in Clear Cell Renal Carcinoma

**DOI:** 10.7150/thno.46825

**Published:** 2020-07-14

**Authors:** Shi-lu Chen, Qun-sheng Huang, Yu-hua Huang, Xia Yang, Ming-ming Yang, Yang-fan He, Yun Cao, Xin-yuan Guan, Jing-ping Yun

**Affiliations:** 1 Sun Yat-sen University Cancer Center, State Key Laboratory of Oncology in South China, Collaborative Innovation Center for Cancer Medicine, Guangzhou 510060, P. R. China.; 2 Department of Pathology, Sun Yat-sen University Cancer Center, Guangzhou 510060, P. R. China.

**Keywords:** clear cell renal carcinoma, GYS1, glycogen, metabolomics, NF-κB

## Abstract

Metabolism reprogramming is a hallmark of many cancer types. We focused on clear cell renal carcinoma (ccRCC) which is characterized by its clear and glycogen-enriched cytoplasm with unknown reasons. The aim of this study was to identify the clinical significance, biological function, and molecular regulation of glycogen synthase 1 (GYS1) in ccRCC glycogen accumulation and tumor progression.

**Methods:** We determined the clinical relevance of GYS1 and glycogen in ccRCC by immunohistochemistry and periodic acid-schiff staining in fresh tissue and by tissue micro-array. Metabolic profiling with GYS1 depletion was performed by metabolomics analysis.* In vitro* and xenograft mouse models were used to evaluate the impact of GYS1 on cell proliferation. High-throughput RNA-Seq analyses and co-immunoprecipitation-linked mass spectrometry were used to investigate the downstream targets of GYS1. Flow cytometry and CCK8 assays were performed to determine the effect of GYS1 and sunitinib on cell viability.

**Results:** We observed that GYS1 was significantly overexpressed and glycogen was accumulated in ccRCC tissues. These effects were correlated with unfavorable patient survival. Silencing of GYS1 induced metabolomic perturbation manifested by a carbohydrate metabolism shift. Overexpression of GYS1 promoted tumor growth whereas its silencing suppressed it by activating the canonical NF-κB pathway. The indirect interaction between GYS1 and NF-κB was intermediated by RPS27A, which facilitated the phosphorylation and nuclear import of p65. Moreover, silencing of GYS1 increased the synthetic lethality of ccRCC cells to sunitinib treatment by concomitantly suppressing p65.

**Conclusions:** Our study findings reveal an oncogenic role for GYS1 in cell proliferation and glycogen metabolism in ccRCC. Re-sensitization of ccRCC cells to sunitinib suggests that GYS1 is a useful indicator of unfavorable prognosis as well as a therapeutic target for patients with ccRCC.

## Introduction

Renal cell carcinoma is one of the most lethal human malignancies worldwide, causing nearly 140,000 deaths per year. Clear cell renal cell carcinoma (ccRCC) is the most common subtype and accounts for approximately 70-80% of RCCs in the urological system. Despite progress in tumor diagnostics and treatment, surgery remains the optimal curative therapy for ccRCC patients. Chemotherapy drugs such as sunitinib, which target vascular endothelial growth factor receptor (VEGFR) and platelet-derived growth factor receptor (PDGFR), are used as the standard of care for ccRCC. Sunitinib, which has been approved by the Food and Drug Administration, is a broad-spectrum small-molecule inhibitor of receptor tyrosine kinases (RTK) and is used as the first-line therapy for advanced ccRCC 1. Although an early response was observed in selected patients, most patients eventually develop resistance to sunitinib. Therefore, it is necessary to improve our understanding of the molecular mechanisms of ccRCC progression to develop better treatment regimens 2.

Dysregulated metabolism is a hallmark of many types of cancer. Tumors reprogram their metabolites to produce sufficient energy for survival. In ccRCC, changes in the central carbon metabolism, amino acid metabolism and glycerophospholipid biochemistry pathways, which endow ccRCC cells with significantly enhanced growth and survival, have recently attracted attention 3, 4. Cells in ccRCC are glycogen-enriched, generating a distinctly clear cytoplasm at the microscopic level. The reason for this enrichment is unknown. Synthesis and breakdown of glycogen involves the activity of several enzymes and regulatory proteins. In kidney tissues, glycogen synthase 1 (GYS1) is the most important rate-limiting enzyme functioning in the last step of glycogen synthesis 5. Pathologically, its deficiency has been shown to cause muscle glycogen storage disease type 0 and death 6, 7. Studies of tumors showed that GYS1 was rapidly induced under hypoxic conditions and positively correlated with glycogen accumulation in glioblastoma, breast, and colon cancer cell lines 8. Aberrant expression of GYS1 not only decreased cellular glycolytic flux but also increased activation of glycogen-responsive AMP kinase, leading to significant growth suppression of myeloid leukemia cells 9. However, the role of GYS1 in ccRCC is still unknown. Because GYS1 is the key enzyme catalyzing glycogen synthesis, we predicted that abnormal expression of GYS1 is involved in manipulating glycogen content and cellular bio-functional transformation in ccRCC.

The nuclear factor NF-κB family includes NF-κB1 (p50), NF-κB2 (p52), RelA (p65), RelB, and c-Rel, which transcriptionally regulate diverse cellular processes 10, 11, including two major pathways which exert different biological roles: the canonical and noncanonical NF-κB pathways. The canonical NF-κB pathway represented by p65 activation has been recognized as the central regulator of tumor progression, whereas the non-canonical NF-κB2 pathway is relatively less well-understood. The NF-κB pathway is an attractive therapeutic target because of its functional correlation with drug sensitivity in many cancer types 12, 13, including sunitinib resistance in ccRCC 14, 15. However, whether the status of the NF-κB pathways is related to the effectiveness of chemo-drugs requires further analysis.

In this study, we focused on the clinical and biological importance of GYS1 in ccRCC and metabolic alterations during this process. Our aim was to investigate GYS1 as a potential oncogene and potential therapeutic target for in ccRCC.

## Material and Methods

### Patients, tissue specimens, and follow-up

A total of 306 patients with primary ccRCC who underwent surgery between January 2004 and December 2012 at Sun Yat-sen University Cancer Center (SYSUCC, Guangzhou, China) were included in this study. The paraffin-embedded tissue samples of these patients were re-embedded into new paraffin blocks for tissue microarray (TMA). This research was approved by the Institute Research Medical Ethics Committee of SYSUCC. All patients provided written informed consent to use of their tissues and data for research purposes. Pathological specimens and pathological data were collected, and all samples were anonymous. The overall survival follow-up period was defined as the interval from the date of surgery to the date of death or last follow-up. No patients had been administered radiotherapy or chemotherapy prior to surgery.

### Hematoxylin-eosin and immunohistochemistry staining

The ccRCC TMA blocks were cut into 4 μm slices and mounted onto glass slides. These slides were then dewaxed and treated with 3% hydrogen peroxide in methanol and blocked using a biotin-blocking kit (DAKO, Glostrup, Germany). Hematoxylin-eosin (HE) and immunohistochemistry (IHC) staining of GYS1 were then conducted and assessed by two independent pathologists who calculated their corresponding IHC score. The scores were determined based on multiplying the staining intensity by the expression proportion. Staining intensity was recorded as four grades (0-3) and proportion was recorded as five grades (0-4) in ascending order of severity. The median IHC score was used as the cut-off value to separate patients into high and low GYS1 expression groups.

### Glycogen detection

PAS staining and quantification assays were conducted using similar protocols as described previously 16. Briefly, the low- and high-PAS staining groups were divided depending on the percentage of positively stained cells. The high-PAS group included >10% positive cells or cluster staining, while sporadic staining or staining area ≤10% were assigned to the low PAS group. Specimens were controlled by diastase PAS staining (D-PAS) to eliminate false-positive cases. The glycogen content in fresh tissues was quantitated with a glycogen assay kit (ab65620, abcam, Cambridge, UK) according to the manufacturer's protocol.

### Cell culture

The ccRCC cells lines Caki-1 and Caki-2 were obtained from the Type Culture Collection Cell Bank, Chinese Academy of Science Committee (Shanghai, China) and were cultured in Dulbecco's modified Eagle's medium (Gibco, Grand Island, NY, USA) supplemented with 10% fetal bovine serum (Gibco, south American). All cells were maintained in a humidified incubator at 37 °C and 5% CO_2_.

### Construction of GYS1 overexpression and knockdown cells

Full-length GYS1 cDNA was cloned into the mammalian vector pENTER. The plasmids were transfected into ccRCC cell lines using Lipofectamine™ 3000 (Invitrogen, Carlsbad, CA, USA) reagent. Small interfering RNAs (siRNAs) and short hairpin RNAs (shRNA) targeting GYS1, NF-κB (p65) and RPS27A were purchased from Shanghai GenePharma Co. Ltd (Shanghai, China). Transfections experiments were performed by using the Lipofectamine™ RNAiMAX (Invitrogen). Puromycin and G418 (Sigma-Aldrich, St. Louis, MO, USA) were used to select stably transduced cells. The siRNAs sequences were listed in Table S1.

### Western Blot

Total cell proteins were extracted using lysis buffer (Beyotime Biotechnology, Shanghai, China) supplemented with protease inhibitor. Western blot was performed based on the previously described standard method 16. Briefly, total protein (30 μg) was loaded in each lane and separated in 10% SDS-PAGE by electrophoresis. The following antibodies against the indicated proteins were used in the immunoblot assays: GYS1 (1:1000, CST, Boston, MA, USA), NF-κB (p65) (1:1000, CST, Boston, MA, USA), p-NF-κB (p-p65) (1:1000, CST, Boston, MA, USA), p-52 (1:1000, CST, Boston, MA, USA), Lamin B1 (1: 500, Santa Cruz Biotechnology, Dallas, TX, USA), β-actin (1:2000, Santa Cruz Biotechnology, Dallas, TX, USA). Band intensities were quantified using the Bio-Rad Molecular Imager ChemiDoc™ XRS+ system.

### RNA extraction and quantitative real-time RT-PCR (qRT-PCR)

Total RNA of fresh tissue and cell lines were isolated and purified using TRIzol reagent (BIOO Scientific Co., Austin, TX, USA). The mRNA was reverse-transcribed into cDNA using standard procedures with Reverse Transcriptase Kits (Vazyme Biotech, Nanjing, China). SYBR green-based quantitative real-time PCR was performed subsequently. The mRNA levels were normalized to the expression of 18S mRNA. The primer sequences used were listed in Table S1.

### Migration assays

After transfection, 5 × 10^4^ cells were harvested and re-plated in the upper compartment of transwell chambers (8 μm pore size, Millipore, Billerica, MA, USA) in serum-free medium. Fresh media containing 10% fetal bovine serum was placed in the lower chamber. The cells were attracted by fetal bovine serum-containing medium in the lower chamber. After incubation for 36 h, the cells in the lower membrane were fixed with methanol for 30 min and then stained with 0.1% crystal violet. Three 20 × magnification fields under a microscope were randomly chosen for counting the cell number.

### *In vivo* tumorigenicity assay

Four-week-old male BALB/c nude mice were purchased from Vital River Company (Beijing, China) and fed under standard conditions. Five mice were randomized into GYS1 overexpression or silencing groups and subcutaneously inoculated with the Caki-1 and Caki-2 cells. Tumor formation was monitored by measuring the tumor volume every 7 days. Four weeks after inoculation, the tumor was harvested, and the tumor volume was calculated as follows: tumor volume (mm^3^) = (length × width^2^)/2. All animal procedures were performed following the SYSUCC Animal Care and Use Committee (IACUC) guidelines.

### Immunofluorescence staining

The cells were seeded into confocal-specific plates. After reaching 50% confluence on the next day, the cells were fixed in 3.7% formaldehyde, permeated with 0.5% Triton X-100, and blocked with 3% bovine serum albumin, followed by incubation with p-p65 or p65 specific primary antibodies overnight or bodipy staining for 30 min. After washing with phosphate-buffered saline three times, the secondary antibody (1:1000, CST, Boston, MA, USA) was added for 1 h incubation to interact with primary antibodies at room temperature. DAPI solution was applied and staining was performed for 5 min. Images were captured using a confocal microscope (Olympus Corporation, Tokyo, Japan).

### Assessment of cell survival and death

For cell activity assays, cultured cells were seeded into 96-well plates. After treatment, the cells were incubated with the Cell Counting Kit-8 (CCK8) reagent for 2 h at 37 °C for 5 consecutive days for proliferation analysis or 48 h for IC_50_ detection. Cell activity was quantified by colorimetric reading in a microplate reader at an absorbance wavelength of 450 nm. For apoptosis analysis, cultured cells were seeded into six-well plates. Cell death was assessed using a Cytotoxicity Apoptosis Detection Kit (Roche, Basel, Switzerland) by flow cytometry with Annexin V/propidium iodide (PI) double staining (Invitrogen). For the colony formation assay, 500 cells were seeded into six-well plates after transfection and cultured in DMEM medium for 10-14 days. The cells were washed with phosphate-buffered saline (PBS) and fixed in methanol for 15 min, followed by staining with 0.1% crystal violet. Cell numbers were counted at the macrographic level. For EdU staining, cultured cells were seeded into 96-well plates after transfection. After 48 h, the cells were fixed in methanol for 15 min, permeated with 0.1% Triton X-100, and blocked with 3% bovine serum albumin before EdU and Hoechst staining. Fluorescence images were captured with a fluorescence microscope (Olympus Corporation, Tokyo, Japan).

### Co-immunoprecipitation and shotgun mass spectrometry

For the co-immunoprecipitation (co-IP) assay, cellular proteins were harvested in lysis buffer (Beyotime Biotechnology, Shanghai, China) and supplemented with a cocktail protease inhibitor. After 10 min of centrifugation at 12,000 rpm to remove the cytoskeleton, specific primary antibody or control rabbit IgG was added to the supernatant and incubated at 4 °C for 4 h. Protein A/G beads (sc-2003, Santa Cruz Biotechnology, Dallas, TX, USA) were then added and incubated at 4 °C overnight. Precipitates were collected by centrifugation at 2000 rpm and washed at least three times with lysis buffer. Interacting proteins were detected by western blotting or shotgun mass spectrometry (MS) (Shanghai Genechem Co., Ltd., China).

### Untargeted metabolomics

Metabolomic profiling was performed in collaboration with Guangzhou Genedenovo Biotechnology Co., Ltd. (Guangzhou, China) including MS analysis and/or bioinformatics analysis. Briefly, Caki-1 cells were treated with scramble or GYS1 siRNAs and dissolved in 1 mL extract solvent (methanol: acetonitrile: water, 2:2:1, v/v) at 36 h post-transfection. A quality control sample was prepared by mixing an equal volume of all samples in each group. The experimental and control groups were tested using six biological duplicates. Liquid chromatography (LC)-MS/MS analyses were performed using an UHPLC system (1290, Agilent Technologies, Santa Clara, CA, USA). MS raw data files were converted to mzML format using ProteoWizard and processed by using the R package XCMS (version 3.2). The variance of metabolites across samples was calculated in positive and negative ion modes according to the LC-MS spectra. Metabolites showing a *P* < 0.05 were considered as differentially expressed metabolites. For statistical analysis, principal component analysis, partial least squares discriminant analysis (PLS-DA), and orthogonal projection to latent structures-discriminant analysis (OPLS-DA) were applied to compare groups using the R package models (http://www.r-project.org/).

### Statistical analysis

Data are shown as means ± standard deviation. Statistical analyses were performed using the SPSS (Version 20.0, IBM, Armonk, NY, USA) and GraphPad PRISM (Version 7.0, GraphPad Software Inc., LaJolla, CA, USA) software. Paired Student's t-test, Pearson's χ2 test, Two-way ANNOVA test, Kaplan-Meier, and multivariate Cox proportional hazard regression models were conducted to analyze independent prognostic factors in overall survival. *P* < 0.05 (two-tailed) was considered statistically significant.

## Results

### GYS1 is upregulated in ccRCC and correlated with poor patient outcomes

**Figure 1:**
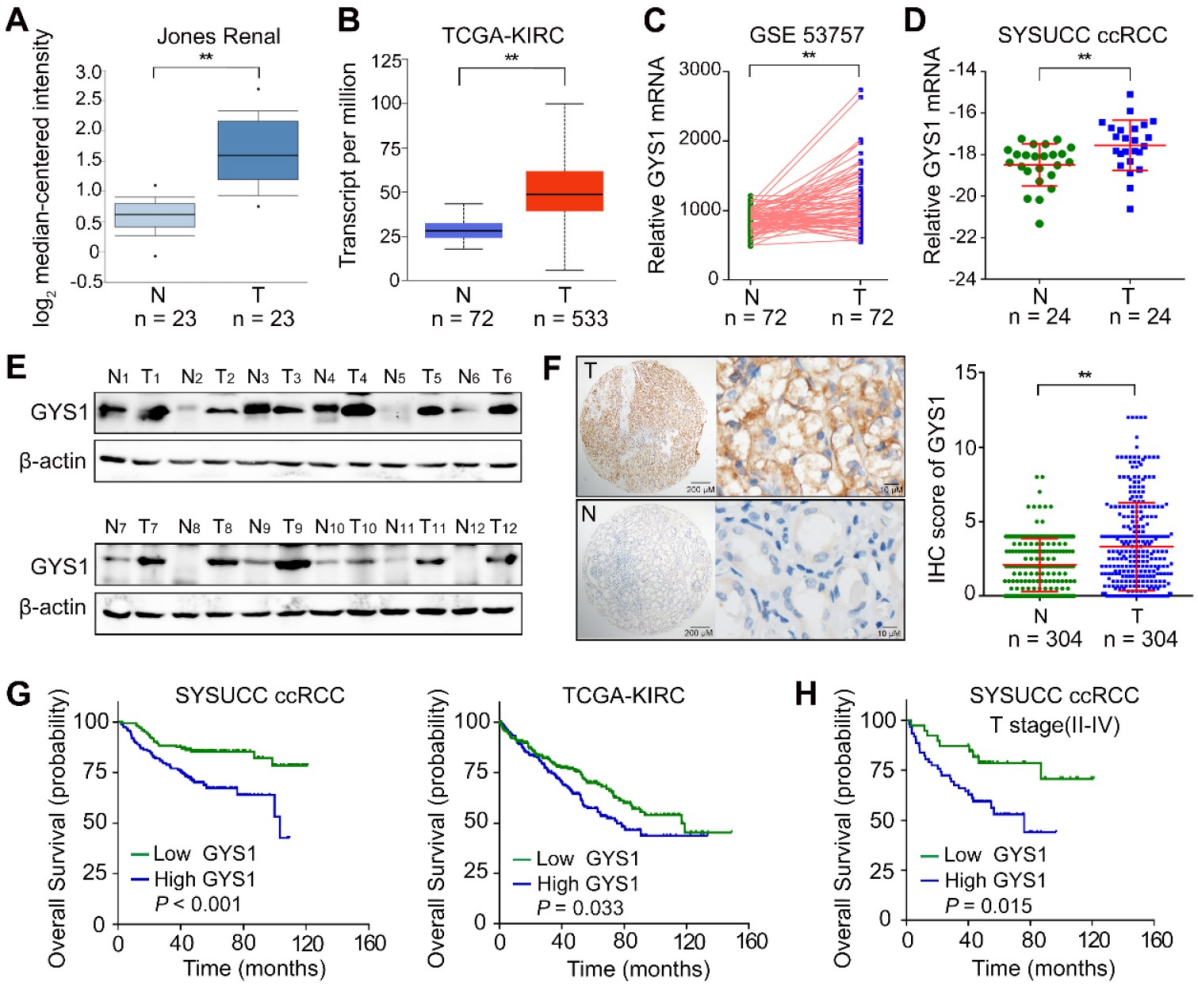
** GYS1 is increased in ccRCC and correlated with poor patient outcome. (A)** The mRNA expression of GYS1 in the Oncomine Jones Renal dataset was determined in tumor (T) and non-tumor (N) cells. **(B)** The mRNA expression of GYS1 in TCGA-KIRC (clear renal cell carcinoma) was determined. **(C)** Expression profile of GYS1 in GEO data GSE53757 was compared. **(D)** GYS1 mRNA levels in 24 paired ccRCC samples were validated using Sun Yat-sen University Cancer Center (SYSUCC) samples. The relative fold-change was normalized with 18S RNA. **(E)** Protein expression of GYS1 was determined by western blotting in 12 paired fresh ccRCC tissue samples. **(F)** Typical IHC images of GYS1 presented in tumor (T) and non-tumor (N) cells. The score of each case is shown in the right.** (G)** Correlation between GYS1 expression and overall survival were determined in the SYSUCC cohort and TCGA cohort by Kaplan-Meier analysis. **(H)** Stratified analysis of T stage (II-IV) patients' overall survival and GYS1 expression. Statistical data are represented as the mean ± SD. **P < 0.01.

To examine the mRNA expression of GYS1 in ccRCC, we first searched the public Oncomine database. We found that GYS1 mRNA expression was significantly increased in tumor tissues compared to in normal tissues in the Jones Renal dataset (Figure 1A). Consistently, elevated GYS1 mRNA was observed in The Cancer Genome Atlas (TCGA) KIRC database (Figure 1B) as well as GEO datasets GSE53757 (Figure 1C). These results were validated in 24 paired ccRCC samples from our center (SYSUCC) (Figure 1D). At the protein level, GYS1 expression was relatively higher in 11 of 12 ccRCC than in matched non-tumor tissues (Figure 1E). Next, a tissue-micro array (TMA) containing 306 ccRCC cases with complete clinicopathological data was evaluated by GYS1 IHC staining (Table S2). GYS1 was found to be primarily located in the cytoplasm. Statistical analysis revealed relatively higher IHC score of GYS1 in tumor samples than in paired non-tumor samples (Figure 1F).

Next, we examined the relationship between GYS1 expression and clinical parameters. High GYS1 expression was statistically associated with age over 55 years (*P* = 0.035), higher Fuhrman nuclear grade (*P* < 0.001), sarcomatoid differentiation (*P* = 0.013), and higher T stage (*P* = 0.038) (Table S3).

To identify the correlation between GYS1 expression and patient outcomes, the median IHC score of GYS1 was selected as the cut-off value to separate ccRCC patients into a high or low expression group. Survival analyses revealed that GYS1 overexpression was significantly correlated with a worse overall survival (OS) in SYSUCC and TCGA patients with ccRCC (Figure 1G). In stratified survival analysis, the high GYS1 group was associated with a shorter OS in the higher T staging cohort (II-IV) (Figure 1H). Univariate and multivariate Cox regression analysis indicated that GYS1 overexpression was an independent prediction factor for OS (*P* < 0.001) (Table S4). Taken together, these results indicated that an increase in GYS1 expression was a prognostic hallmark for ccRCC.

### Metabolic profiling is altered with the depletion of GYS1

**Figure 2:**
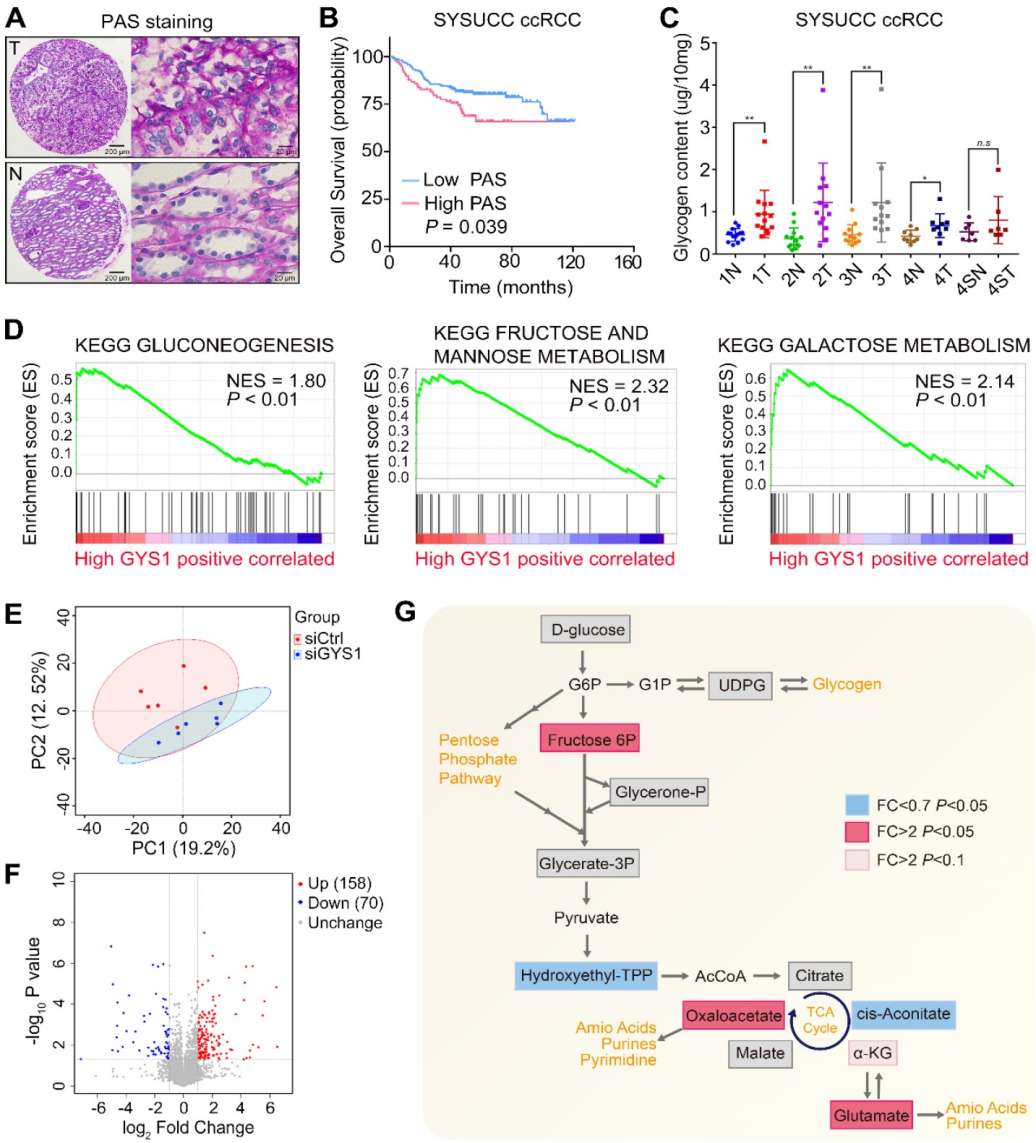
** Metabolic alterations in glycogen in ccRCC-associated GYS1 expression. (A)** Representative images of tumor (T) and non-tumor (N) with PAS staining in ccRCC TMA. **(B)** Correlation of PAS staining and overall survival was determined by Kaplan-Meier analysis.** (C)** Glycogen levels were measured by quantitative colorimetry in ccRCC and corresponding adjacent non-tumor tissues (Fuhrman Grade 1: 1N & 1T; Fuhrman Grade 2: 2N & 2T; Fuhrman Grade 3: 3N & 3T; Fuhrman Grade 4 without sarcomatoid differentiation: 4N & 4T; Fuhrman Grade 4 with sarcomatoid differentiation: 4SN & 4ST). **(D)** GSEA revealed that high GYS1 expression was correlated with three KEGG glucose metabolism pathways. **(E)** Principal component analysis (PCA) of metabolomics data. Color of dots indicate control (red) and GYS1 knockdown (blue) samples. **(F)** Volcano plot profiling of all annotated metabolites. There were 158 increased and 80 decreased metabolites showing significantly different abundance (*P* < 0.05) compared to in the GYS1 silencing and control groups. **(G)** Metabolic changes in central carbon metabolism in metabolomics data. Metabolites are labeled as color-coded squares. Color corresponds to the up (red) or down (blue) expression in the GYS1-silenced group. Gray denotes detected metabolites without significant changes. Pink denotes tendency to upregulation with *P* < 0.1. Statistical data are represented as **P < 0.01.

Recently ccRCC metabolism has attracted renewed interest based on integrative analysis of transcriptomic and metabolomic data 3. In this regard, we assessed whether the glycogen content was changed in ccRCC samples compared with corresponding non-tumor renal tissues by conducting PAS staining. D-PAS was also detected as a control to avoid false-positive staining (Figure 2A & Figure S1A). Compared to paired non-tumor tissues, glycogen levels within the tumor samples were significantly increased and correlated with poor patient survival (Figure 2B & Figure S1B). A glycogen detection kit further supported the increased glycogen levels in the tumors compared with paired non-tumor tissues, except for the Furhman grade 4 samples with sarcomatoid differentiation. Furthermore, there were no statistical differences between tumors in different grades (Figure 2C). By analyzing the TCGA-KIRC project based on the Gene Set Enrichment Analysis (GSEA), we found that the glycolysis, fructose, and galactose metabolism pathways were positively enriched in the high GYS1 expression group, revealing that carbohydrate metabolism was markedly altered by elevated expression of GYS1 in cancer (Figure 2D).

As GYS1 was known to catalyze glycogen synthesis, we detected metabolomic perturbations in Caki-1 cells with GYS1 silencing by untargeted metabolomics. MS revealed a total of 628 differential peaks in positive and negative ion modes totally, which matched 228 identifiable metabolites (Figure S1C). Principal component analysis (PCA), PLS-DA, and OPLS-DA revealed clear separation between the control and GYS1 knock-down groups (Figure 2E & Figure S1D). A volcano map revealed 158 increased and 70 decreased metabolites (*P* < 0.05) (Figure 2F). The glucose downstream metabolite fructose 6-phosphate was significantly increased in Caki-1 cells. Five TCA cycle intermediate metabolites were measured, which revealed a dichotomous pattern of changes with an elevated level of oxaloacetate and decreased in the transient intermediate product cis-aconitate. α-Ketoglutarate tended to be higher in GYS1-silenced cells although not significantly (fold-change = 5.73, *P* = 0.051). We also observed a relatively large increase in glutamate, which was consistent with the previous findings that TCA flux alteration activated anaplerotic glutamine flux and amino acid content via broad shifts from central carbon metabolism to amino acid metabolism 3, 17 (Figure 2G).

To further explore whether GYS1 plays a role in lipid metabolism, we analyzed the correlation of GYS1 expression with lipid storage and synthesis. We found that overexpressed or silenced GYS1 did not influence lipid content in Caki-1 or Caki-2 cells (Figure S2A). FAS activity detection and qRT-PCR assays. Results showed that overexpressed or silenced GYS1 did not influence lipid content in Caki-1 and Caki-2 cells (Figure S2A). Fatty acid synthase activity was not associated with GYS1 in ccRCC cells (Figure S2B). The mRNA expression of the lipid storage and synthesis enzymes FAS, CPT1A, and SERBP1 were not changed (Figure S2C). These results indicated that GYS1 expression did not directly correlated with lipid metabolism in ccRCC.

### GYS1 promotes ccRCC cell proliferation both *in vitro* and *in vivo*

**Figure 3:**
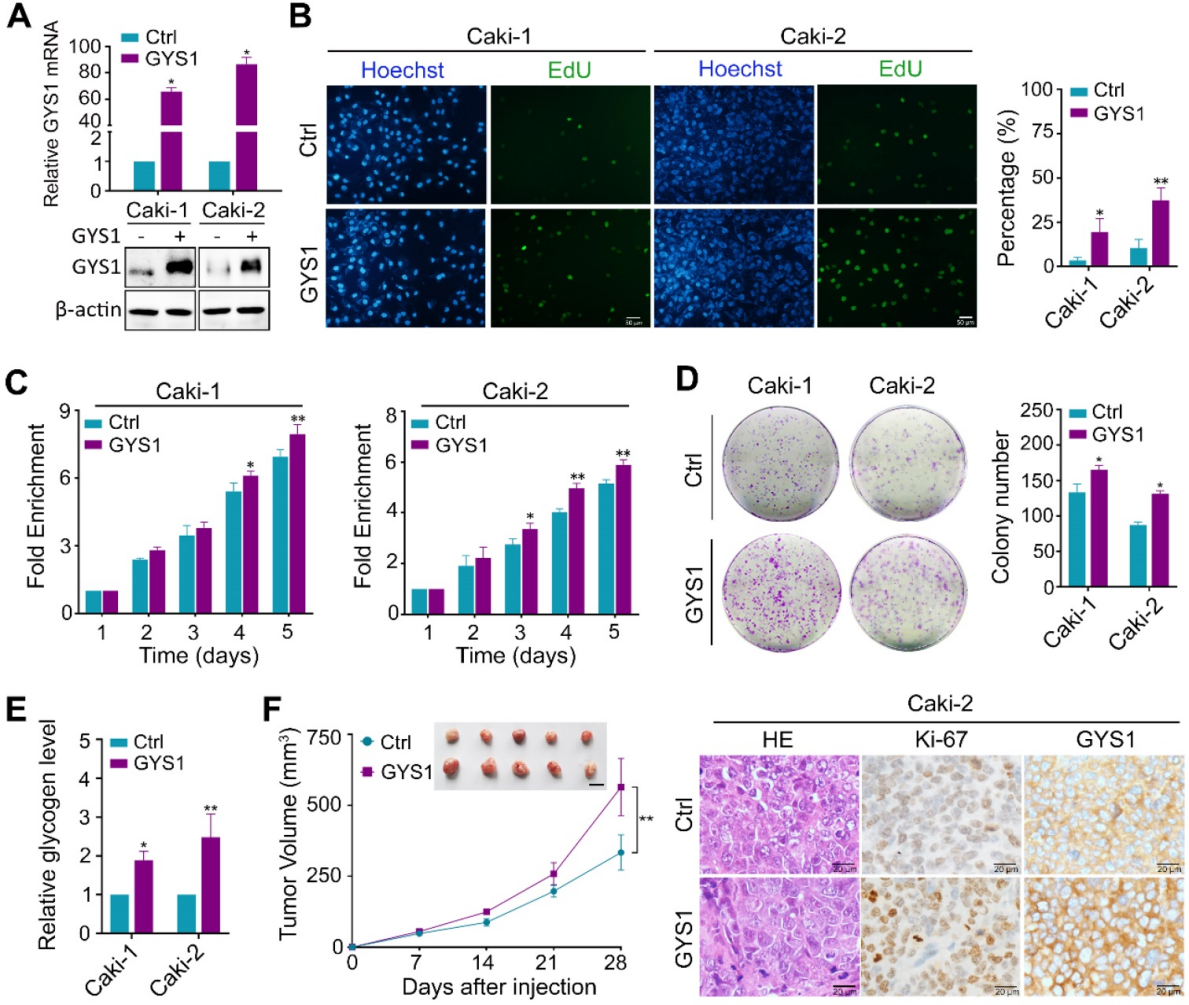
** GYS1 promotes ccRCC proliferation *in vitro* and *in vivo.* (A)** GYS1 was overexpressed by transfection with GYS1 exogenous vector in Caki-1 and Caki-2 cells. The mRNA and protein levels of GYS1 were determined by qRT-PCR and western blotting. **(B)** EdU assays showed the replication of DNA in cells induced by GYS1. Green staining denotes duplicated cells and blue denotes the cell nucleus.** (C)** Cell activity was detected by CCK8 assay over five consecutive days. Relative absorbance was measured at OD_450_. Fold-enrichment was normalized to the absorbance on day 1. **(D)** Colony formation assays to determine the effect of GYS1 on cell growth. The number of colonies was counted using ImageJ software (NIH, Bethesda, MD, USA). **(E)** The glycogen content in cell lines with GYS1 overexpression were detected by a quantitative glycogen detection kit.** (F)** Xenograft mice experiment to evaluate tumor growth* in vivo*. Mice were sacrificed at 28 days after inoculation with Caki-2 cells. The volume of tumors in each group was calculated. Representative images of HE staining and IHC are shown. Statistical data are represented as the mean ± SD. *P < 0.05, **P < 0.01.

Next, we focused on the biological function of GYS1 in ccRCC cells. We explored the effects of GYS1 overexpression by transfecting an ectopic-expressing vector into the Caki-1 and Caki-2 cell lines (Figure 3A). Forced expression of GYS1 increased cell proliferation capacity and colony formation ability compared to in the scrambled control according to EdU staining, CCK8, and colony formation assays (Figure 3B-D). Upon GYS1 overexpression, the glycogen content in ccRCC cells increased (Figure 3E). In contrast, silencing of GYS1 by two mixed siRNAs in Caki-1 and Caki-2 attenuated cell growth with concomitantly reduced glycogen content in ccRCC cells (Figure S3A-E). However, overexpression or silencing of GYS1 did not affect cell migration as detected by Transwell assays (Figure S4). The effects of GYS1 on cell proliferation were further corroborated in xenograft models. Caki-1 and Caki-2 cells transfected with GYS1 overexpression vector grew faster with stronger staining of the proliferation marker Ki67 (Figure 3F & Figure S5A). In contrast, GYS1 silencing reduced the tumor growth rate (Figure S3F & Figure S5B). Based on these results, GYS1 promoted ccRCC cell proliferation both* in vitro* and *in vivo*.

### Functional and pathway enrichment analysis of GYS1-related genes in ccRCC

**Figure 4:**
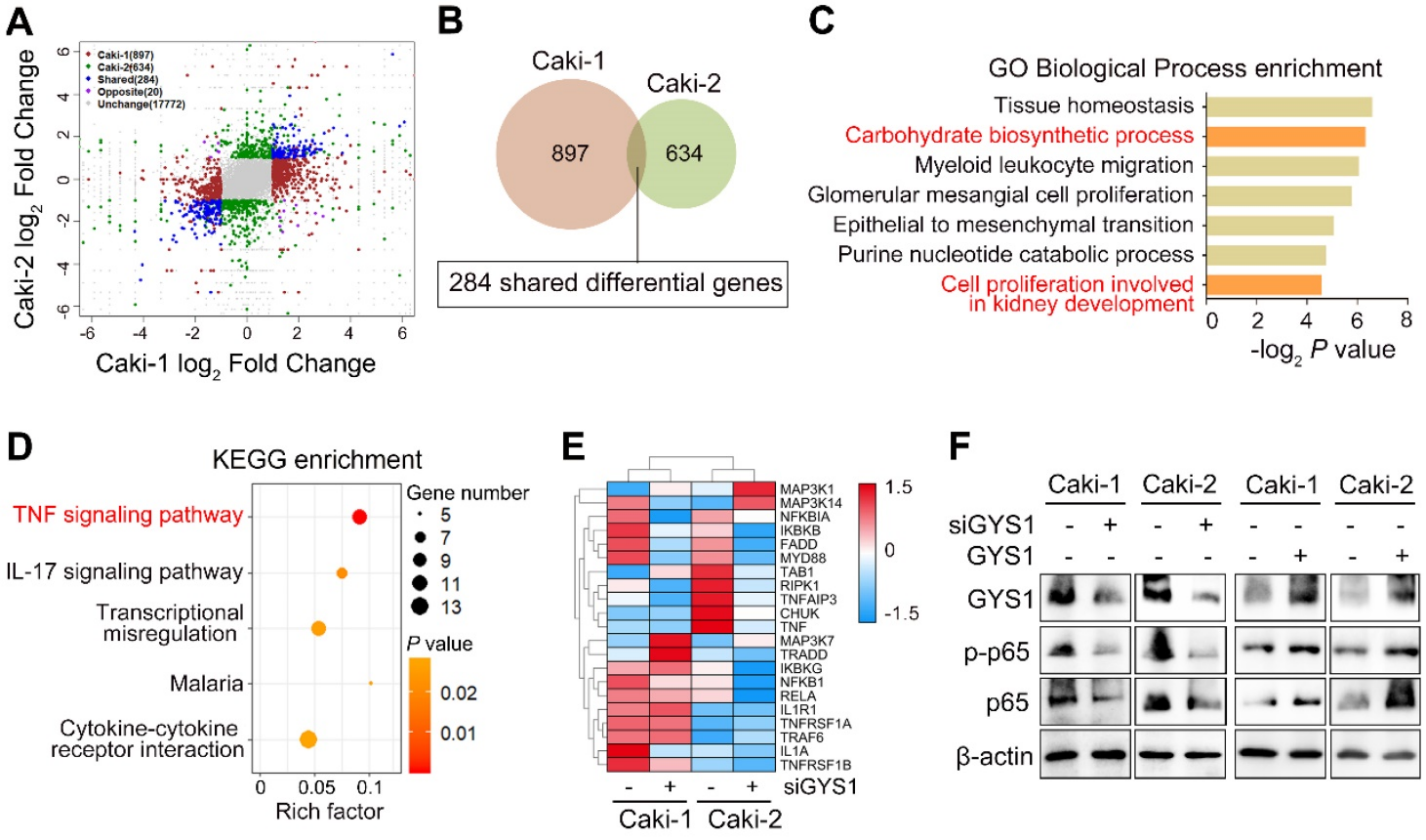
** Pathway-based analysis of GYS1 depletion by RNA sequencing. (A)** Clustering of transcriptome sequencing in Caki-1 and Caki-2 cells with GYS1 siRNA transfection. Significance was set based on *P* < 0.05 and absolute fold-change > 2. **(B)** Venn diagraph filtered 284 shared differential genes in Caki-1 and Caki-2 cells with GYS1 silencing. **(C)** Gene Ontology (GO) enrichment analysis indicated the biological processes influenced by the filtered genes. **(D)** KEGG enrichment of filtered genes indicated signaling transduction pathway alterations.** (E)** Heatmap was generated based on BIOCARTA NF-κB pathway clustering. **(F)** Western blot indicated the expression of p-p65 and p65 regulated by GYS1.

To further dissect the functions and molecular mechanisms of GYS1 in ccRCC progression, we performed transcriptome profiling by RNA-sequencing in Caki-1 and Caki-2 cells transfected with GYS1 siRNAs. Based on the filtering conditions of false discovery rate (FDR) < 0.05 and |log_2_FC|>1, 897 and 634 genes were differentially expressed in Caki-1 and Caki-2 cells respectively, among which 284 genes were mutually up- or downregulated in both cell lines (Figure 4A-B). Gene ontology (GO) analysis of these shared genes showed biological processes enrichment in carbohydrate biosynthetic processes and cell proliferation involved in kidney development (Figure 4C). KEGG enrichment analysis revealed that tumor necrosis factor-α (TNF-α) signaling pathways were markedly influenced by GYS1 (Figure 4D). A heatmap showed that the expression of a series of genes in the BIOCARTA NF-κB pathway were altered (Figure 4E). According to previous studies, canonical NF-κB (p65/RELA) was the pivotal pathway involved in TNF-α stimulation. We found that overexpression of GYS1 increased, whereas GYS1 knockdown decreased total p65 and phosphorylated p65 (p-p65) (Figure 4F). Thus, the Caki-1 and Caki-2 cell lines might share similar mechanisms of cell growth through the cascade response triggered by the canonical NF-κB pathway.

### GYS1 promotes p65 phosphorylation and nuclear accumulation

**Figure 5:**
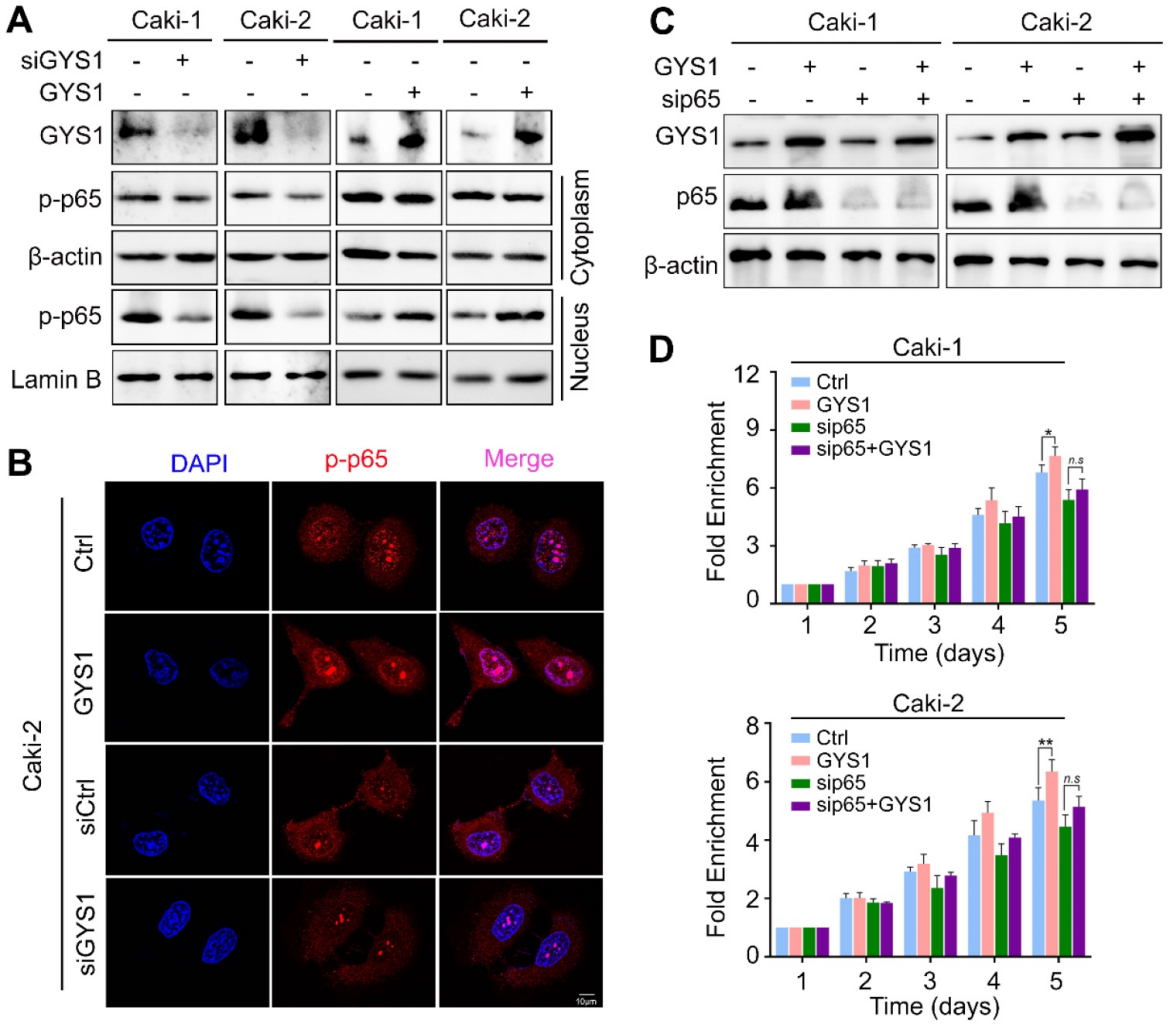
** GYS1 activates the NF-κB signaling pathway. (A)** Western blot revealed the expression of p-p65 in the cytoplasm and nucleus. β-Actin and Lamin B were used as internal references in the cytoplasm and nucleus, respectively. **(B)** IF images indicating the subcellular localization of p-p65 (red). The nuclear outline was stained by DAPI (blue) in Caki-2 cells. **(C)** Expression of p65 and p-p65 was measured by western blotting following transfection of p65 siRNA in rescue experiments. **(D)** Cell viability was assessed by CCK8 in rescue experiments. Statistical data are represented as the mean ± SD. *P < 0.05, **P < 0.01.

Since p-p65 was activated by its nuclear accumulation, we separated the cytoplasmic and nuclear protein to determine whether p-p65 was relocated by GYS1.The p-p65 nuclear accumulation was observed with GYS1 overexpression whereas GYS1 knockdown reduced nuclear proportion of p-p65 (Figure 5A). These results were supported by those of IF staining (Figure 5B and Figure S6).

To further test whether p65 activation was required for the downstream effect of GYS1 on cell proliferation, we conducted *in vitro* rescue experiments. Following depletion of p65 by transfection of siRNAs, the function of GYS1 on cell proliferation was reduced, as shown by CCK8 assays (Figure 5C-D). These results demonstrate that GYS1 promoted cell growth in the presence of p65.

### GYS1 depends on interact of RPS27A with p65

**Figure 6:**
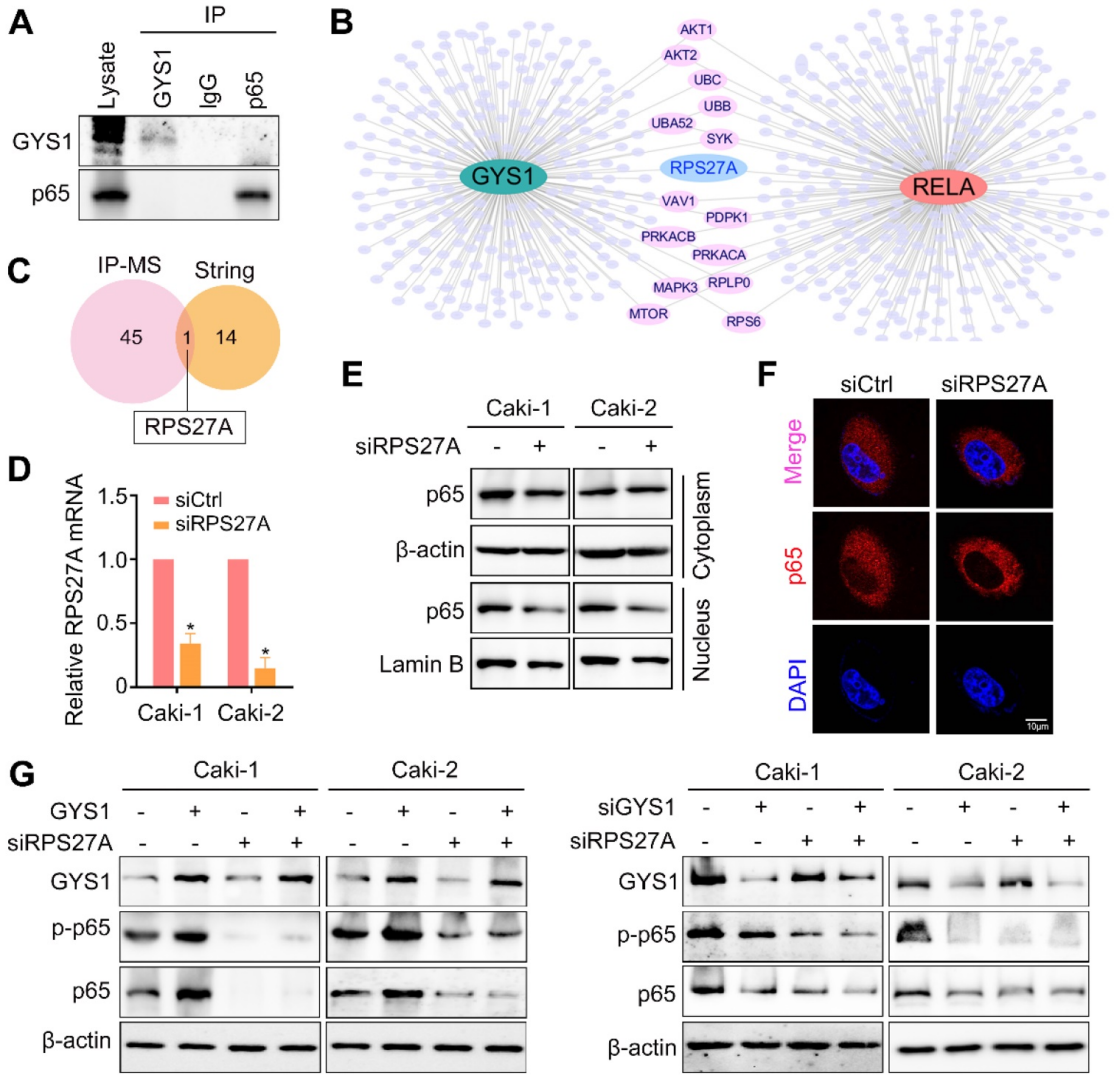
** GYS1 regulates p65 via RPS27A. (A)** Co-immunoprecipitation assays detected no interaction between GYS1 and p65. Cell lysate was immunoblotted with anti-GYS1 and anti-p65. **(B)** STRING database revealed the protein mediating the interaction between GYS1 and p65 (RELA). **(C)** Venn diagram indicating that RPS27A was identified by both IP-mass spectrum and the STRING database. **(D)** The silencing effect of RPS27A siRNAs was validated by qRT-PCR. **(E)** Western blot revealed the expression of p-p65 in the cytoplasm and nucleus. β-Actin and Lamin B were used as internal references in the cytoplasm and nucleus, respectively.** (F)** IF images indicating the subcellular localization of p65 (red). The nuclear outline was stained by DAPI (blue) in Caki-2 cells.** (G)** Expression of p65 and p-p65 was measured by western blotting following GYS1 overexpression or silencing in RPS27A-depleted cells. Statistical data are represented as the mean ± SD. *P < 0.05.

To gain insight into how GYS1 regulates p65 nuclear import, we carried out co-IP to detect their interacting proteins. However, mass spectrum did not identify direct binding of these two proteins (Figure 5A). Thus, the effect of GYS1 on p65 might be mediated by a chaperonin. Several studies have indicated that although p65 typically forms dimers mainly with p50, other small proteins may act as chaperonins to regulate the functions of this complex. We searched a series of intermediated proteins linking GYS1 and p65 according to the mass spectrum data. Combining the protein-protein interaction network data shown in the STRING database, ribosomal protein S27a (RPS27A) was found to be the potential protein intermediate between GYS1 and p65 (Figure 6B-C).

Ribosomal proteins (RPs) are abundant RNA-binding proteins with multiple functions. To determine the functions of RPS27A besides its canonical role in intermediating the interaction between GYS1 and p65, we used siRNAs to silence the expression of RPS27A (Figure 6D). As shown by western blotting and IF staining, the nuclear localization of p65 was inhibited by reduced RPS27A expression (Figure 6E-F). The effect of GYS1 on p65 expression was also abolished in the absence of RPS27A (Figure 6G). These results suggest that RPS27A was necessary for the regulation of p65 by GYS1.

### GYS1-depletion reverses sunitinib resistance of ccRCC cells

**Figure 7:**
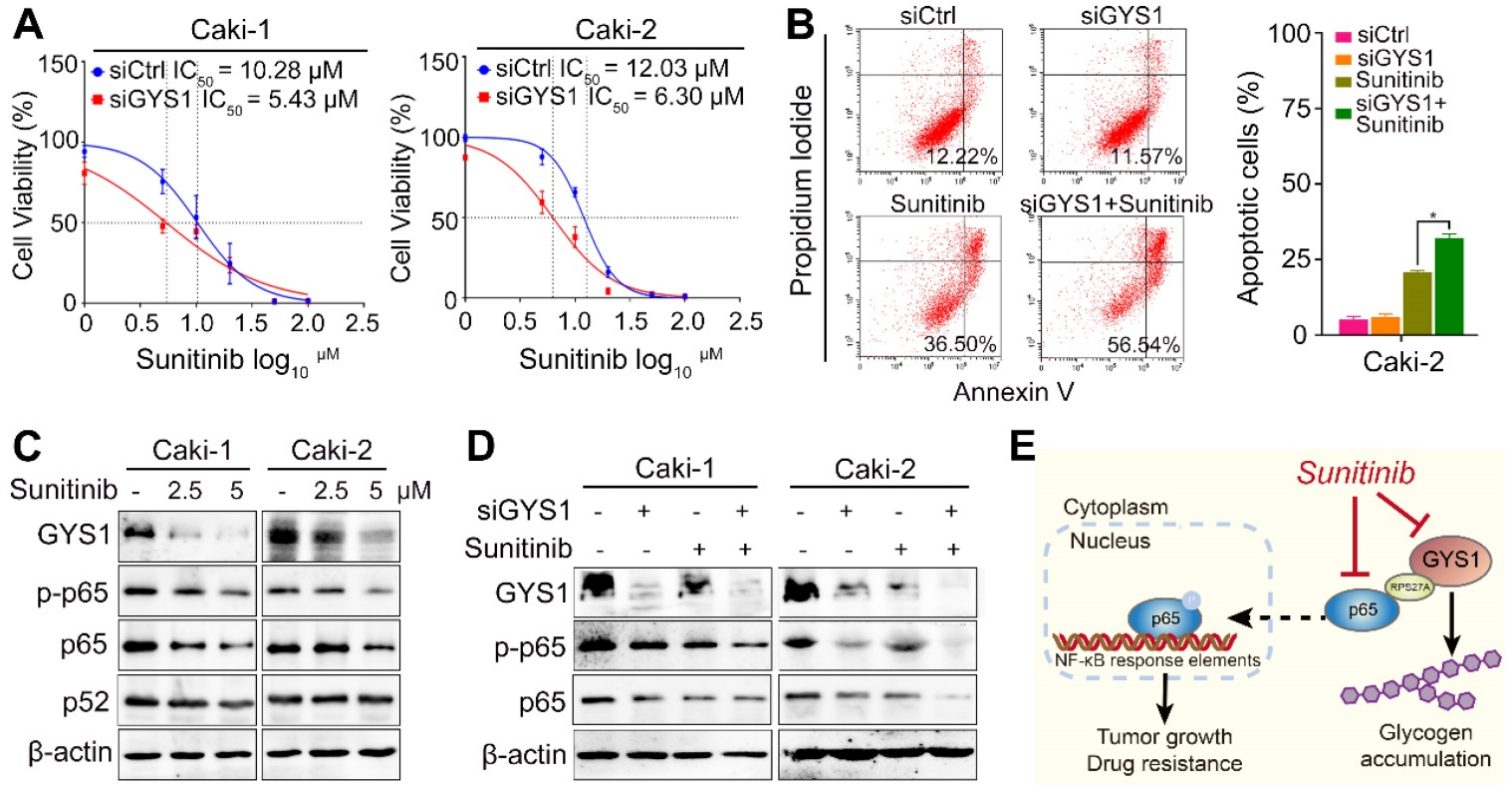
** GYS1 is synergistic with sunitinib in ccRCC cells. (A)** Cell viability was detected by CCK8 assay at 48 h after sunitinib treatment. IC_50_ was calculated using GraphPad Prism software (GraphPad, Inc., La Jolla, CA, USA). **(B)** Flow cytometry showed apoptotic cells by Annexin V/PI double staining in Caki-2 cells. **(C)** Western blotting revealed the protein expression of the GYS1 and NF-κB pathways 24 h after sunitinib treatment. **(D)** Protein level of p65 and p-p65 was measured after treatment with GYS1 siRNAs and sunitinib. **(E)** Schematic diagram showing the molecular regulation mechanisms in our study. Statistical data are represented as the mean ± SD. *P < 0.05.

Drug resistance has been one of major limitations in ccRCC treatment. To evaluate the influence of GYS1 on the response of ccRCC to chemotherapy, cell viability was measured in the presence and absence of sunitinib in GYS1-silenced Caki-1 and Caki-2 cells. The GYS1-silenced groups were more sensitive to treatment with sunitinib, showing a nearly two-fold difference in the IC_50_ compared with control groups (Figure 7A), suggesting that intrinsic GYS1 was sufficient and responsible for ccRCC cells' sensitivity to sunitinib. Considering that the induction of apoptosis is one of major mechanisms inducing cell death by sunitinib, we further investigated the pro-apoptotic effect in response to sunitinib treatment and GYS1 silencing. As expected, the percentage of apoptosis induced by sunitinib treatment was increased following GYS1 knockdown in Caki-2 cells (Figure 7B). These results indicate that the combination of targeting GYS1 and sunitinib treatment was effective for treating patients with ccRCC.

Because NF-κB was reported to be corelated with drug resistance in RCC 18, we predicted that the activity of p65 could explain why GYS1 and sunitinib have a synergistic effect on cell apoptosis. Treatment with sunitinib reduced the expression of GYS1 and p65 rather than p52 in a dose-dependent manner, as demonstrated by western blotting (Figure 7C), suggesting that the canonical NF-κB pathway accounted for cell apoptosis. Synchronous treatment by GYS1 silencing and sunitinib significantly reduced the expression of p65 and p-p65 compared to each single treatment (Figure 7D). This synthetic lethality was largely abolished by inhibition of p65 (Figure S7). Taken together, these results suggest GYS1 as a potential therapeutic target for patients with ccRCC showing sunitinib resistance (Figure 7E).

## Discussion

GYS1 is the key enzyme in the last step of glycogen synthesis. Its deficiency is known to cause glycogen storage disease type 0 and death 6, 7. Overexpression of GYS1 was associated with adverse outcome in acute myeloid leukemia 19 and non-small cell lung cancer 20 in a small cohort of patients. However, we still lack sufficient evidence whether GYS1 serves as a key potential hallmark of cancer. Since ccRCC is the most common lethal subtype accounting for renal carcinoma with unfavorable outcome compared with papillary renal cell carcinoma, and chromophobe renal carcinoma, it is essential to examine the clinical significance of GYS1 in large human samples. By using TMA containing 306 ccRCC patients, we revealed a correlation between increased GYS1 expression and unfavorable patient outcome, higher Fuhrman nuclear grade, higher T stage, and sarcomatoid differentiation, which pinpointed GYS1 as an independent potential prognostic biomarker of ccRCC.

The metabolic reprogramming of ccRCC has attracted attention in recent years 21. However, extensive studies have focused on glycolysis and gluconeogenesis in ccRCC, while abnormalities of glycogen deregulation have not been widely examined. Although the abnormal expression of several glycolytic genes, including phosphoglycerate kinase 1 (PGK1), lactate dehydrogenase A (LDHA), and pyruvate dehydrogenase kinase 1 (PDK1) 22 as well as glycogen phosphorylases PYGL 23 and gluconeogenic enzyme fructose-1,6-bisphosphatase 1 (FBP1) 17 have been reported in ccRCC, the underlying molecular mechanism of glycogen deregulation remains elusive. A number of observations have suggested a critical role of glycogen in the survival of cancer cells. For example, inhibition of glycogen breakdown could induce apoptosis in pancreatic tumors 24, and glycogen content in breast, kidney and ovary cancer cell lines inversely correlated with proliferation 25. Furthermore, we have demonstrated that glycogen synthase 2 (GYS2) depletion leads to downregulation of hepatocellular carcinoma glycogen and accelerate tumor proliferation 16. Although glycogen detected by PAS staining showed an inverse correlation with patient outcome, it was not an independent factor showed strong correlation with clinical parameters (Table S5 &S6). Furthermore, the Fuhrman grade 4 ccRCC samples with sarcomatoid differentiation did not showed statistical differences between tumor and non-tumor samples (Figure 2C). One explanation to these results was the the genetic landscape differences between sarcomatoid RCC (sRCC) and ccRCC. For example, a higher mutation rate of TP53 (42.3%) was detected in sRCC, compared with only 2% TP53 mutation in ccRCC 26. In this regard, the molecular alterations of sarcomatoid differentiation might be distinctive to low grade ccRCC. Since 74.5% (228/306) of the patients in our cohort have lower Fuhrman grading (Grade1 and grade2), we speculate that GYS1 might be more important for the glycogen accumulation in the early stage ccRCC.

Glycogen synthesis and breakdown involve multiple steps regulated by a series of enzymes and regulatory proteins, and it is likely that other factors (e.g., PYGL, HIF1α and GLUT1) participate in ccRCC glycogen regulation. Although direct links between these genes and glycogen content have not been tested in ccRCC, there are some hints shown in previous data. For example, PYGL catalyzes the key step of glycogen degradation, and its depletion leads to consequent glycogen accumulation in cancer cells 8. The abnormal expression of PYGL is also observed in ccRCC 23. Hypoxia is known as a crucial condition for glycogen accumulation under hypoxic conditions, and has been observed in U87 glioblastoma, MCF-7 breast, and HCT116 colon cancer cells 8. HIF1α and HIF2α regulate glycogen accumulation and acquisition of a clear cell phenotype in VHL deficiency, independently of glycogenolysis and glycogen synthesis enzymes 27. GLUT1, which facilitates the uptake and incorporation of glucose to glycogen, was found to be increased by HIF-1α in VHL-deficient ccRCC 28 and was synergistically overexpressed with GYS1 and PYGL in proliferating cells 29. Considering the complexity of glycogen regulatory networks, it is necessary to conduct further studies for the understanding of increased glycogen in ccRCC.

Metabolic reprogramming has been implicated in the development and progression of tumors and can even render tumors vulnerable to chemotherapy 30, 31. Interestingly, during rewiring of glucose metabolism, cancer cells show corresponding changes in their chemo- or radio-resistance 31. Consistently with this, overexpression of GYS1 is associated with adverse outcomes and a poor response to azacitidine in myelodysplastic syndromes and acute myeloid leukemia 19. The effect of synthetic lethality by silencing of GYS1 together with sunitinib treatment observed in our study highlights the therapeutic potential of targeting GYS1 and improves the current understanding of glucose metabolism-related drug resistance. We also detected concomitant suppression of p65 rather than p52 during sunitinib treatment, and thus the canonical NF-κB pathway status should be considered in the clinical prediction of the therapeutic response of patients with ccRCC. Since sunitinib is a receptor tyrosine kinases (RTK) inhibitor, RTKs may be an additional factor accounting for sunitinib resistance. In phospho-RTK analysis, the feedback reactivation of several kinases induced by sunitinib were suppressed after combination treatment with GYS1 siRNAs in Caki-2 cells. This indicates that the combination effect of GYS1 and sunitinib, at least in part, also accounts for the effects of RTKs (Figure S8).

The most abundant and canonical form of NF-κB is the p65-p50 heterodimer complex which regulates immunity, inflammation, and apoptosis. In addition to p50, various molecules interact with p65 to modulate its functions. Indirect modification between GYS1 and p65 is mediated by the multifunctional proteinencoded by the ubiquitin carboxyl extension protein 80 (UBCEP80) gene 32. The N-terminal domain of UBCEP80 codes for mono-ubiquitin whereas the C-terminal region codes for a carboxyl extension protein, which eventually becomes a part of the mature 40S ribosomal subunit 33. RPS27A was overexpressed in actively proliferating cells, including chronic myeloid leukemia 34, colon 35, renal 36, and breast cancers 37. RPS27A also performs extra-ribosomal functions when ribosome synthesis is perturbed. For example, RPS27A regulates LMP1 protein expression encoded by EBV in a proteasome-dependent pathway 38. When shuttled from the nucleolus into the nucleoplasm, RPS27A enhanced p53 stability by suppressing MDM2-mediated p53 ubiquitination in response to ribosomal stress. Binding of RPS27A to MDM2 frees more p53 for the activation of p21^Waf1^, which in turn can induce cell cycle arrest and cell death 39, 40. In addition to RPS27A, another ribosomal protein, RPS3, intervenes in NF-κB-mediated transcription by selectively recruiting p65 to certain promoters, further validating the importance of ribosomal proteins in oncogenic pathways regulation besides their canonical roles 41.

One limitation of our study was insufficient direct evidence demonstrating glycogen accumulation and ccRCC progression. We did not determine whether glycogen was used as an energy source for tumor growth, as it is difficult to reduce the cytoplasmic glycogen content bypass-modulating genes. In addition, glycogen storage and GYS1 expression differ between paired tumor and non-tumor samples in ccRCC, papillary renal cell carcinoma, and chromophobe renal carcinoma (Figure S9). Whether ccRCC and chromophobe renal carcinoma share similar tumorigenesis mechanisms manifested by elevated cellular glycogen, beyond the situation in papillary renal cell carcinoma, remains unclear. Further studies are needed to determine these details.

In summary, our study demonstrated the significance of elevated glycogen and GYS1 in ccRCC. We highlighted the functional importance of GYS1 in mediating ccRCC progression. These data complement our current understanding of this cancer's genotype and phenotype, and reveal promising potential drug targets for ccRCC patients.

## Supplementary Material

Supplementary figures and tables.
