# Supplementary material for: GYS1 induces glycogen accumulation and promotes tumor progression via the NF-κB pathway in Clear Cell Renal Carcinoma
Source: Theranostics. 2020 Jul 14;10(20):9186–99. doi: 10.7150/thno.46825 (PMC7415807; doi:10.7150/thno.46825)
Supplement: Supplementary file 1 — Supplementary figures and tables. [file thnov10p9186s1.pdf]

Supplementary Tables

Table S1. Primers and siRNAs

Primers

Identifier Type	Sequence (5’-3’)
GYS1-F	CAGCGCGGACCAACAATTTC
GYS1-R	TCCTCCCGAACTTTTCCTTCA
RPS27A-F	CTGGAAGATGGACGTACTTTGTC
RPS27A -R	CGACGAAGGCGACTAATTTTGC
18S-F	TGAGAAACGGCTACCACATCC
18S-R	ACCAGACTTGCCCTCCAATG
FAS-F	GAAGAGTCTTCGTCAGCCAGGA
FAS-R	TGGCTTCATAGGTGACTTCCA
SREBP1-F	ACTTCTGGAGGCATCGCAAGCA
SREBP1-R	AGGTTCCAGAGGAGGCTACAAG
CPT1A-F	TCCAGTTGGCTTATCGTGGTG
CPT1A-R	TCCAGAGTCCGATTGATTTTTGC

siRNAs

Identifier Type	Sequence (5’-3’)
GYS1-1	CCAACGACGCUGUCCUCUUTT
GYS1-2	CCAUCGAGGCACAGCACUUTT
RPS27A-1	TTAGTCGCCTTCGTCGAGA
RPS27A-2	CAGACATTATTGTGGCAAA
p65-1	GAUGAGAUCUUCCUACUGU
p65-2	GCCCUAUCCCUUUACGUCA

Table S2. The characteristics of patients diagnosed with ccRCC in SYSUCC in 2004–2012 (n = 306)

Characteristic	Number	Percentage (%)
Age(years)		
≤55	174	56.9
>55	132	43.1
Gender		
Male	209	68.3
Female	97	31.7
Fuhrman grading		
1	46	15.0
2	182	59.5
3	59	19.3
4	19	6.2
Sarcomatoid differentiation		
No	292	95.4
Yes	14	4.6
Necrosis		
No	228	74.5
Yes	78	25.5
Vascular invasion		
No	280	91.5
Yes	26	8.5
T stage		
I	205	67.0
II	64	20.9
III	29	9.5
IV	8	2.6
Lymph nodes invasion		
No	283	92.5
Yes	23	7.5

ccRCC, clear cell renal cell carcinoma; SYSUCC, Sun Yat-sen University Cancer Center.

Table S3. Correlation between GYS1 expression and clinicopathological features in ccRCC (n = 304)

Variable	All cases	GYS1 expression		*P* value
		Low	High	
Age(years)				
≤55	172	90(52.3%)	82(47.7%)	**0.035**
>55	132	53(40.2%)	79(59.8%)	
Gender				
Male	208	99(47.6%)	109(52.4%)	0.775
Female	96	44(45.8%)	52(54.2%)	
Fuhrman grading				
1-2	226	123(54.4%)	103(45.6%)	**0.000**
3-4	78	20(25.6%)	36(74.4%)	
Sarcomatoid differentiation				
No	290	141(48.6%)	149(51.4%)	**0.013**
Yes	14	2(14.3%)	12(85.7%)	
Necrosis				
No	227	114(50.2%)	113(49.8%)	0.056
Yes	77	29(37.7%)	48(62.3%)	
Vascular invasion				
No	278	133(47.8%)	145(52.2%)	0.359
Yes	26	11(38.5%)	15(61.5%)	
T stage				
I	203	104(51.2%)	99(48.8%)	**0.038**
II-IV	101	39(32.4%)	62(67.6%)	
Lymph nodes invasion				
No	282	135(47.9%)	147(52.1%)	0.298
Yes	22	8(36.4%)	14(63.6%)	

Table S4. Univariate and multivariate analyses of overall survival in ccRCC (n=305)

	Univariate analysis		Multivariate analysis	
	Hazard ratio (95% CI)	*P* value	Hazard ratio (95% CI)	*P* value
Age(years＞55)	1.934(1.223-3.057)	**0.005**	1.523(0.945-2.453)	0.084
Gender(male)	1.008(0.619-1.641)	0.974		
Furman nuclear grade(3-4)	3.017(1.911-4.763)	**0.000**	1.555(0.859-2.815)	0.144
Sarcomatoid differentiation	5.958(3.036-11.692)	**0.000**	1.785(0.773-4.117)	0.175
Necrosis	2.648(1.674-4.189)	**0.000**	1.316(0.783-1.045)	0.356
Vascular invasion	2.428(1.276-4.618)	**0.007**	1.078(0.508-2.291)	0.845
T stage(Stage II-IV)	2.285(1.452-3.595)	**0.000**	1.314(0.773-2.233)	0.312
Lymph nodes invasion	4.344(2.464-7.657)	**0.000**	2.504(1.296-4.838)	**0.006**
GYS1(High)	2.437(1.484-4.003)	**0.000**	1.783(1.045-3.042)	**0.035**

Table S5. Correlation between PAS expression and clinicopathological features in ccRCC (n = 305)

Variable	All cases	PAS staining		*P* value
		Low	High	
Age(years)				
≤55	174	119(68.4%)	55(31.6%)	0.822
>55	131	88(67.2%)	43(32.8%)	
Gender				
Male	208	142(68.3%)	66(31.7%)	0.826
Female	97	65(67.0%)	32(33.0%)	
Fuhrman grading				
1-2	227	157(69.2%)	70(30.8%)	0.409
3-4	78	50(64.1%)	28(35.9%)	
Sarcomatoid differentiation				
No	291	200(68.7%)	91(31.1%)	0.143
Yes	14	7(50.0%)	7(50.0%)	
Necrosis				
No	227	159(70.0%)	68(30.0%)	0.165
Yes	78	48(61.5%)	30(38.5%)	
Vascular invasion				
No	279	194(69.2%)	85(30.8%)	**0.041**
Yes	26	13(50.0%)	13(50.0%)	
T stage				
I	205	142(69.3%)	63(30.7%)	0.454
II-IV	100	65(65.0%)	35(35.0%)	
Lymph nodes invasion				
No	282	192(68.1%)	90(31.9%)	0.777
Yes	23	15(65.2%)	18(34.8%)	

Table S6. Univariate and multivariate analyses of overall survival in ccRCC (n = 305)

	Univariate analysis		Multivariate analysis	
	Hazard ratio (95% CI)	*P* value	Hazard ratio (95% CI)	*P* value
Age (years > 55)	1.934(1.223-3.057)	**0.005**	1.681(1.047-2.697)	**0.032**
Gender (male)	1.008(0.619-1.641)	0.974		
Furman nuclear grade (3-4)	3.017(1.911-4.763)	**0.000**	1.989(1.133-3.493)	**0.017**
Sarcomatoid differentiation	5.958(3.036-11.692)	**0.000**	1.925(0.848-4.370)	0.117
Necrosis	2.648(1.674-4.189)	**0.000**	1.113(0.617-2.009)	0.721
Vascular invasion	2.428(1.276-4.618)	**0.007**	0.920(0.432-1.959)	0.829
T stage (Stage II-IV)	2.285(1.452-3.595)	**0.000**	1.354(1.021-1.796)	**0.036**
Lymph nodes invasion	4.344(2.464-7.657)	**0.000**	2.208(1.158-4.209)	**0.016**
PAS (High)	1.623(1.020-2.581)	**0.041**	1.388(0.856-2.251)	0.183

Supplementary Figures

A

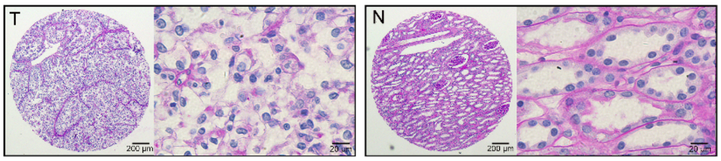

B

GYS1	PAS Low	PAS High
Low	113	29
High	93	68

P < 0.001

C D

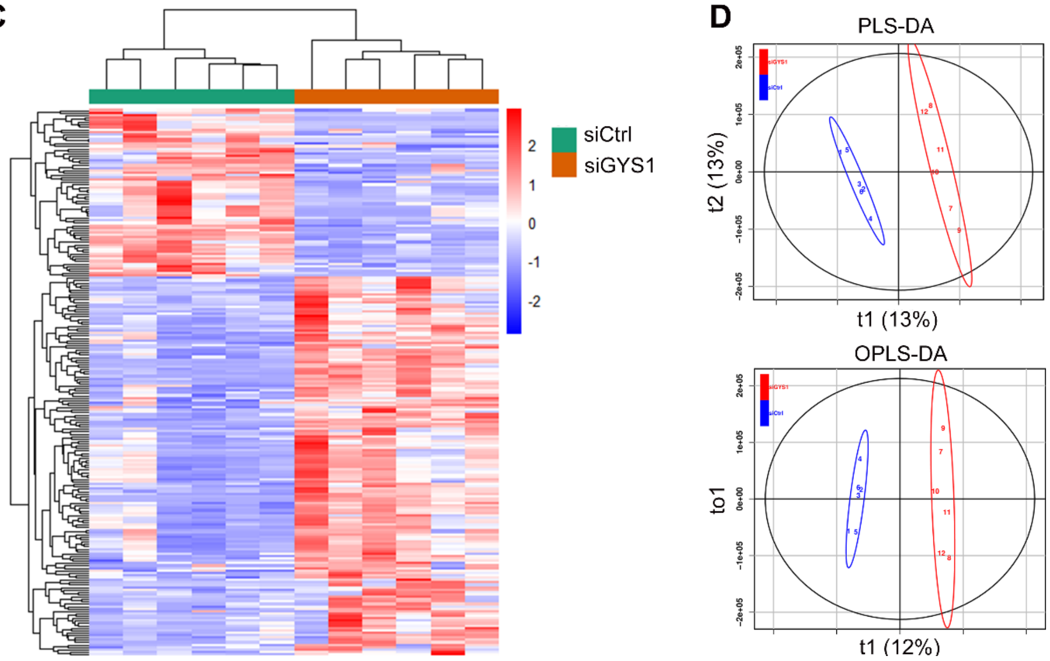

Figure S1. Metabolites alterations in ccRCC cells. (A) Representative images of tumor (T) and non-tumor (N) with D-PAS staining in ccRCC TMA. **(B)** The correlation of PAS staining and GYS1 expression in TMA tumor was determined by chi-square analysis. **(C)** Heatmap clustering of altered metabolites (P < 0.05) in Caki-1 cells with GYS1 siRNAs treatment. **(D)** PLS-DA and OPLS-DA analysis of metabolomics data. Color of dots indicated control (blue) and GYS1 knockdown (red) samples.

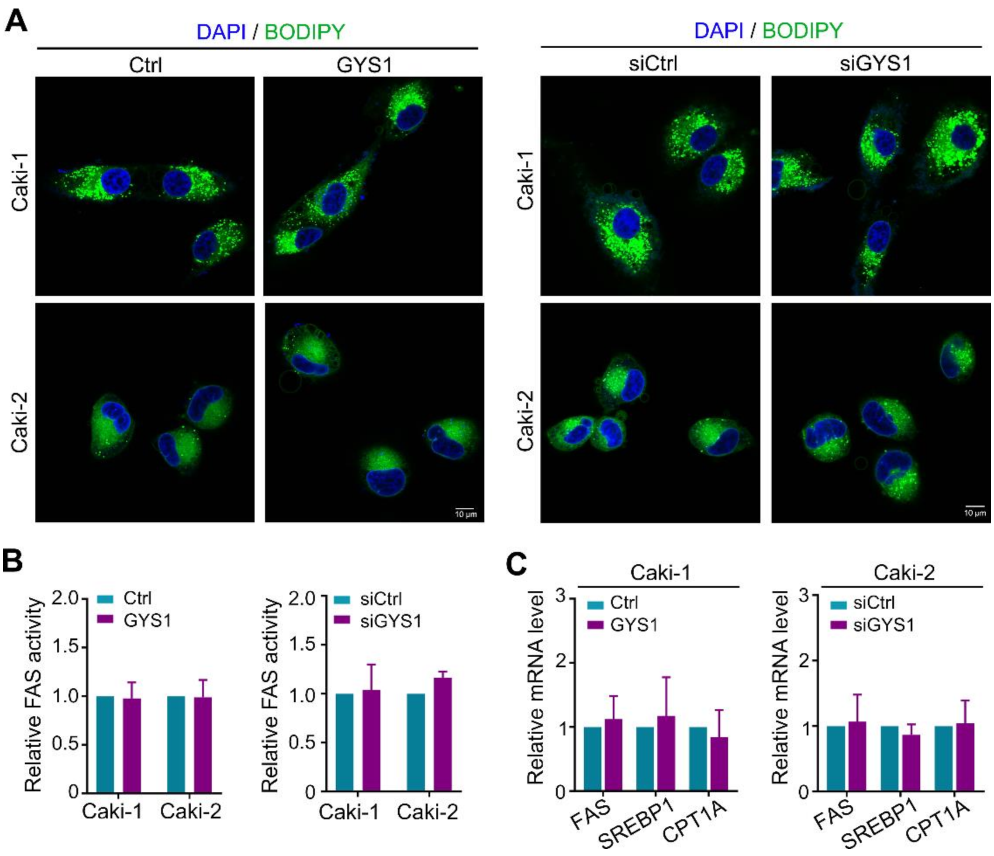

Figure S2. GYS1 has no effect on lipid metabolism. (A) Lipid content was measured by BODIPY immunofluorescent staining in ccRCC cells. **(B)** FAS activity was detected by a commercial kit in ccRCC cells. **(C)** The mRNA expression of the lipid storage and synthesis enzymes FAS, CPT1A and SERBP1 were examined by RT-PCR.

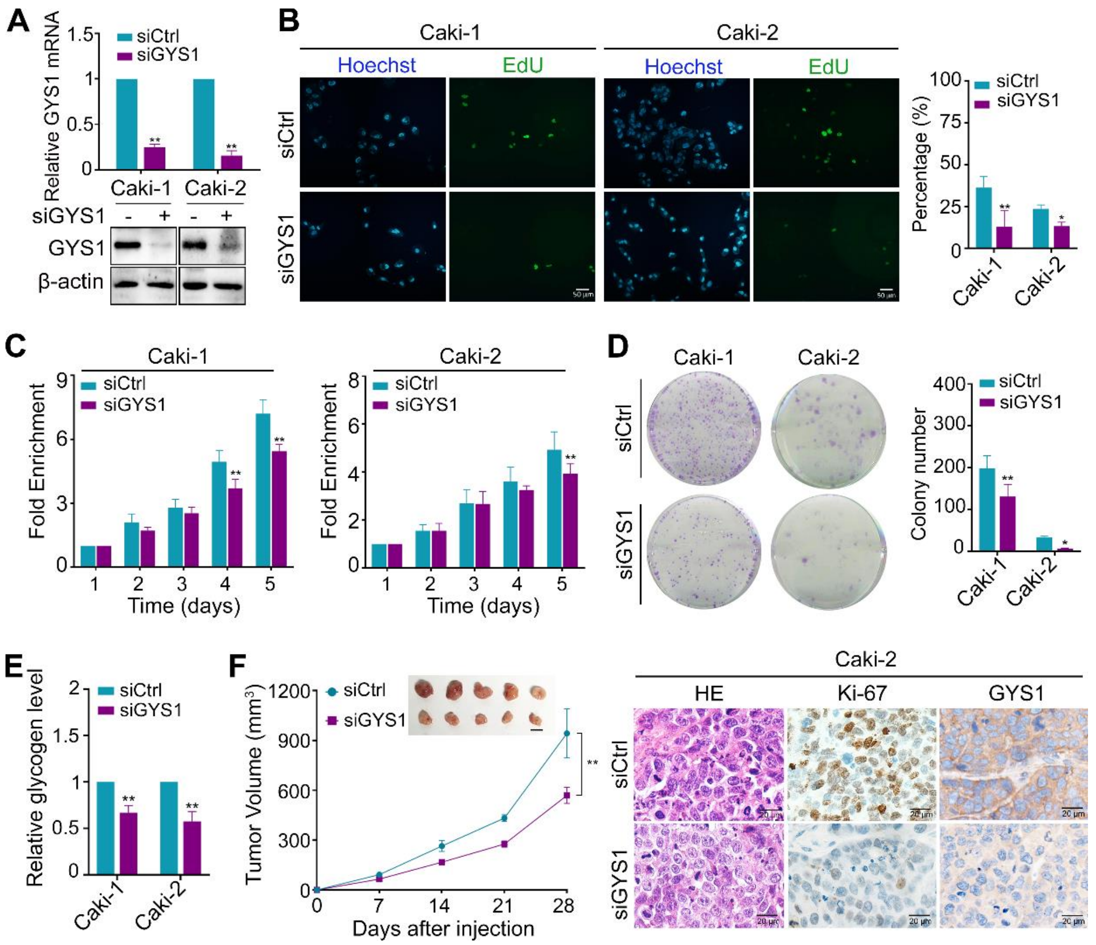

Figure S3. GYS1 inhibition suppresses ccRCC proliferation *in vitro* and *in vivo.* **(A)** GYS1 was depleted by transfection with siRNAs in Caki-1 and Caki-2 cells. The mRNA and protein levels of GYS1 were determined by qRT-PCR and western blot. **(B)** EdU assays showed the replication of DNA in cells induced by GYS1. Green staining denotes duplicated cells and blue denotes cell nucleus. **(C)** Cell activity was detected by CCK8 assay over five consecutive days. Relative absorbance was measured at OD_{450}. Fold enrichment was normalized to the absorbance record on day 1. **(D)** Colony formation assays to determine the effect of GYS1 on cell growth. The number of colonies were counted using Image J software. **(E)** The glycogen content in cell lines with GYS1 silencing were detected by quantitative glycogen detection kit. **(F)** Xenograft mice experiment to evaluate the tumor growth *in vivo*. Mice were sacrificed at 28 days after inoculation. The volume of tumors was calculated. Representative images of HE staining and IHC are shown. Statistical data are represented as mean ± SD. **P* < 0.05, ***P* < 0.01.

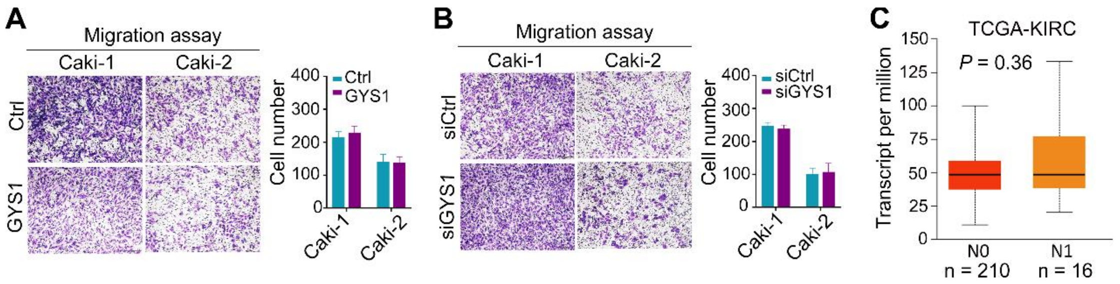

Figure S4. GYS1 has no effect on metastasis. (A-B) Representative images of transwell migration assay by GYS1 overexpression (A) or silencing (B). **(C)** TCGA-KIRC data showed no GYS1 expression difference between cases with or without lymph node metastasis. (N0 = No regional lymph node metastasis; N1 = Metastases 1-3 axillary lymph nodes).

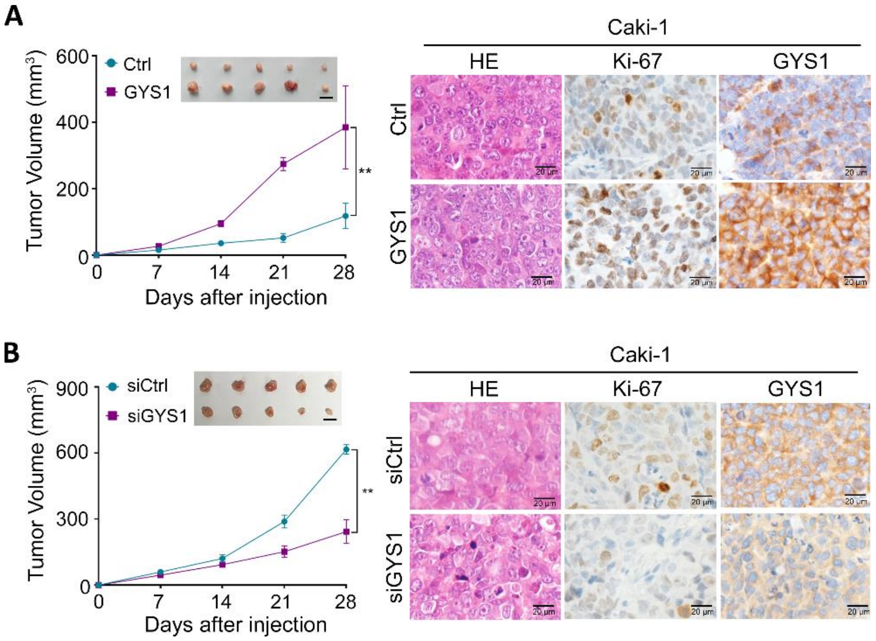

Figure S5. Xenograft mice experiment to evaluate the tumor growth *in vivo*. Caki-1 cells with GYS1 overexpression **(A)** or silencing **(B)** was injected into both flanks of the mice. Mice were sacrificed at 28 days after inoculation. The volume of tumors was calculated. Representative images of HE staining and IHC are shown. Statistical data are represented as mean ± SD. $^{**}P < 0.01$.

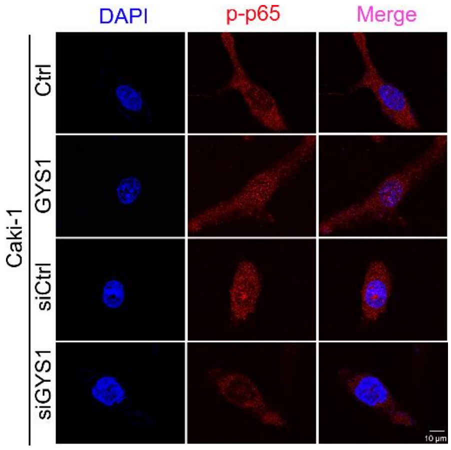

Figure S6. GYS1 activates NF-κB signaling pathway. IF images indicating the subcellular localization of p-p65 (red) referred to nucleus outlined stained by DAPI (blue) in Caki-1 cells.

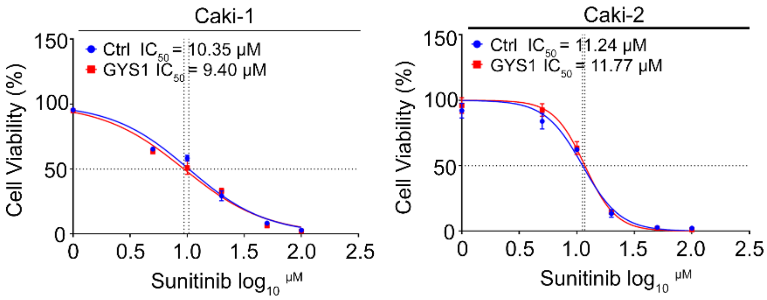

Figure S7. GYS1 is synergistic with sunitinib in ccRCC cells. Cells were treated by p65-depletion by transfection with p65 siRNAs. Then cell viability was detected by CCK8 assay at 48 h after GYS1 silencing and sunitinib treatment. IC_{50} was calculated using GraphPad Prism software.

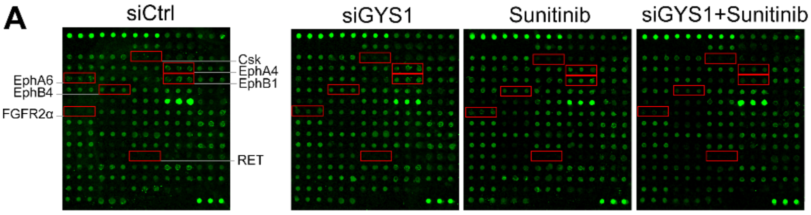

B

RTKs	siGYS1	Sunitinib	siGYS1+Sunitinib
Csk	2.09	2.07	1.10
ErbB4	3.88	2.50	1.71
EphA4	1.68	3.14	1.31
FGFR2 (α isoform)	3.80	3.42	1.26
EphB1	3.19	4.20	0.96
RET	48	65	49

Fold Change Normalized to siCtrl

Figure S8. RTK activation detection. (A) The detection of receptor tyrosine kinases (RTK) activity was detected by protein microarray. **(B)** The list of altered kinases was shown in the table.

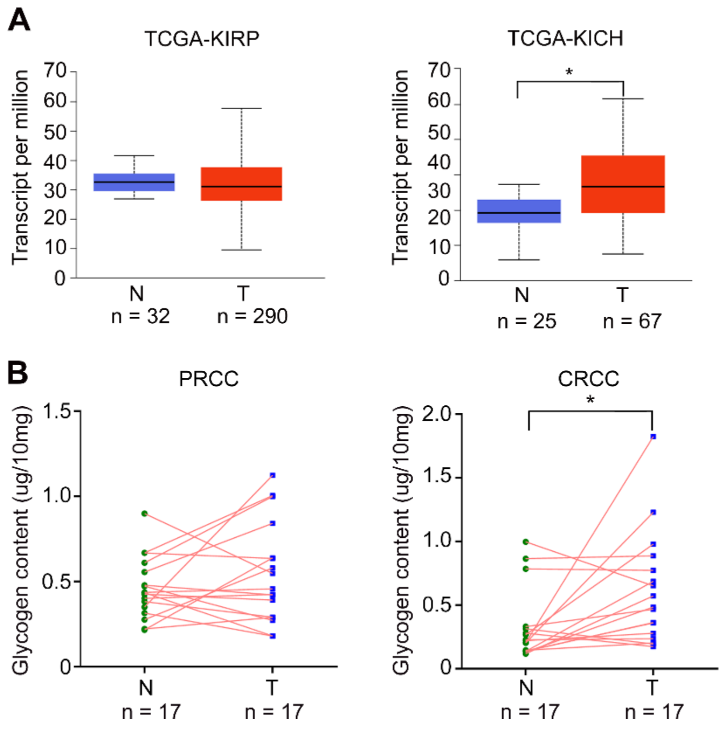

SFigure S9. GYS1 and glycogen expression in renal carcinoma. (A) mRNA expression of GYS1 in TCGA-KIRP (papillary renal cell carcinoma, PRCC) and TCGA-KICH (chromophobe renal cell carcinoma, CRCC) were determined. **(B)** Glycogen levels were measured by quantitative colorimetric method in 17 ccRCC and corresponding adjacent non-tumor tissues. *$P < 0.05$.
